# Diagnostic performance of radiomics in predicting axillary lymph node metastasis in breast cancer: A systematic review and meta-analysis

**DOI:** 10.3389/fonc.2022.1046005

**Published:** 2022-11-28

**Authors:** Xiuru Gong, Yaxin Guo, Tingting Zhu, Xiaolin Peng, Dongwei Xing, Minguang Zhang

**Affiliations:** Shanghai Municipal Hospital of Traditional Chinese Medicine, Shanghai University of Traditional Chinese Medicine, Shanghai, China

**Keywords:** breast cancer, axillary lymph node metastasis, radiomics, meta-analysis, review

## Abstract

**Background:**

This study aimed to perform a meta‐analysis to evaluate the diagnostic performance of radiomics in predicting axillary lymph node metastasis (ALNM) and sentinel lymph node metastasis (SLNM) in breast cancer.

**Materials and methods:**

Multiple electronic databases were systematically searched to identify relevant studies published before April 29, 2022: PubMed, Embase, Web of Science, Cochrane Library, China National Knowledge Infrastructure, and Wanfang Data. The quality of the included studies was assessed using the Quality Assessment of Diagnostic Accuracy Studies-2 tool. The overall diagnostic odds ratio (DOR), sensitivity, specificity, and area under the curve (AUC) were calculated to evaluate the diagnostic performance of radiomic features for lymph node metastasis (LNM) in patients with breast cancer. Spearman’s correlation coefficient was determined to assess the threshold effect, and meta-regression and subgroup analyses were performed to explore the possible causes of heterogeneity.

**Results:**

A total of 30 studies with 5611 patients were included in the meta-analysis. Pooled estimates suggesting overall diagnostic accuracy of radiomics in detecting LNM were determined: DOR, 23 (95% CI, 16-33); sensitivity, 0.86 (95% CI, 0.82-0.88); specificity, 0.79 (95% CI, 0.73-0.84); and AUC, 0.90 (95% CI, 0.87-0.92). The meta-analysis showed significant heterogeneity between sensitivity and specificity across the included studies, with no evidence for a threshold effect. Meta-regression and subgroup analyses showed that combined clinical factors, modeling method, region, and imaging modality (magnetic resonance imaging [MRI], ultrasound, computed tomography [CT], and X-ray mammography [MMG]) contributed to the heterogeneity in the sensitivity analysis (*P* < 0.05). Furthermore, modeling methods, MRI, and MMG contributed to the heterogeneity in the specificity analysis (*P* < 0.05).

**Conclusion:**

Our results show that radiomics has good diagnostic performance in predicting ALNM and SLNM in breast cancer. Thus, we propose this approach as a clinical method for the preoperative identification of LNM.

## Introduction

Female breast cancer has recently surpassed lung cancer to become the most common cancer worldwide (1). Increasingly young patients develop breast cancer, among which axillary lymph node metastasis (ALNM) is the most common mode of metastasis. Axillary lymph nodes (ALN) reside in the axillary region of the upper limb and carry important information for the anatomic staging of breast cancer. The presence and extent of axillary lymph node metastasis reflect the risk of distant recurrence and death after topical treatment. Moreover, the overall survival rate of node-positive patients is 40% lower than node-negative (2, 3). Thus, the lymph node status is crucial for treatment planning, surgical procedures, and prognosis assessment of patients (4).Currently, staging of the axilla in patients with breast cancer is evaluated with sentinel lymph node biopsy and axillary lymph node dissection. However, both involve invasive surgery and have a high incidence of complications (5–7). Therefore, developing noninvasive ALN staging methods for determining the clinical stage of breast cancer and selecting individualized treatment options for patients is paramount.

Radiomics is a novel noninvasive method that rapidly extracts numerous quantitative features imperceptible to the naked eye from traditional biomedical images through high-throughput computation. It provides valuable diagnostic and prognostic information by analyzing the correlation between image features and clinical data, which is widely used in tumor grading, therapeutic response, and prognostic assessment (8, 9).In breast cancer, radiomics constructs predictive models for ALNM and SLNM by extracting quantitative features from ultrasound, computed tomography (CT), magnetic resonance imaging (MRI), X-ray mammography (MMG), or positron emission tomography (PET), with good potential (10–14). However, due to differences in study methods and imaging modalities, heterogeneity may arise among the studies. Moreover, in patients with breast cancer, systematic research on the performance of radiomics in predicting lymph node metastasis (LNM) and the factors affecting it is lacking, necessitating further verification of radiomics for clinical application.

Therefore, the objective of this systematic review with meta-analysis was to evaluate the diagnostic performance of radiomics models in predicting ALNM and SLNM in patients with breast cancer.

## Materials and methods

The meta-analysis was conducted according to the Preferred Reporting Items for Systematic Reviews and Meta-analysis (PRISMA) Statement (15).

### Literature search

Two observers independently searched various electronic databases: PubMed, Embase, Web of Science, Cochrane Library, China National Knowledge Infrastructure, and Wanfang Data to identify eligible studies published before April 29, 2022. “Breast Neoplasms,” “Lymphatic Metastasis,” “Radiomics,” and their variations were used as search terms. Only English and Chinese publications were sought, and EndNote 20 software was used for reference management. Disagreement among the observers, if any, was resolved with a consensus achieved through discussion with the third observer.

### Selection criteria

The inclusion criteria were as follows:

1. Original research studies
2. Studies on patients with breast cancer and ALNM or SLNM, according to pathological criteria
3. Radiomics studies, including ultrasound, CT, MRI, MMG, or PET–CT images applied for ALNM or SLNM classification
4. Data sufficient to reconstruct the 2 × 2 contingency table to estimate the sensitivity and specificity of the diagnosis

The exclusion criteria were as follows:

1. Reviews, editorials, expert opinions, animal studies, and conference presentations
2. Imaging analysis based on only non-radiomics methods
3. Unclear pathological diagnosis
4. Studies with smaller sample sizes, if involving overlapping patients and data

### Data extraction

Relevant data were extracted from the included studies: the first author, publication year, study design, country, sample size, the number of LNM and non-LNM in breast cancer, reference standard, imaging modalities, radiomics algorithm, and clinical factors. True-positive, false-positive, false-negative, and true-negative values were extracted from the data, and the 2×2 contingency table was generated. When multiple models were used for a patient group, the one with the highest diagnostic accuracy was selected for the meta-analysis. Disagreement was discussed until consensus was reached.

### Data quality assessment

The risk of bias in the selected studies was assessed using the 4 areas of the Quality Assessment of Diagnostic Accuracy Studies-2 (QUADAS-2) tool: patient selection, index test, reference standard, and flow and timing, customized to a particular study question (16).

### Statistical analysis

Statistics were calculated using the Midas modules in the Stata software (v 16.0) and Review Manager (v 5.3). The pooled sensitivity, specificity, diagnostic odds ratio (DOR), positive and negative likelihood ratios (LR), with corresponding 95% confidence intervals (CIs), were determined to predict diagnostic accuracy. The summary receiver operating characteristic (SROC) curve and the area under the curve (AUC) were also constructed using the random effects model to evaluate the diagnostic value of combined studies (17). The AUC values suggested the discriminatory power as follows: low, 0.5-0.7; moderate, 0.7-0.9; and high, > 0.9.

Heterogeneity between the included studies was assessed with a Cochran Q test and the *I^2^* statistics. A difference was considered significant when *P* < 0.05, with *I^2^* ≥ 50% indicating a moderate-to-high heterogeneity among the studies. Pooling of studies and effect size were evaluated using a random-effect model, suggesting the distribution of true effects among the heterogenous studies (18). The causes of heterogeneity were estimated with threshold effect and meta-regression analyses. Spearman’s correlation coefficient was calculated to assess the threshold heterogeneity, with *P* < 0.05 implying a threshold effect (19). Univariable meta-regression analysis of several relevant covariates: study design (retrospective or prospective), combined clinical factors (yes or no), modeling method (radiomic algorithm or machine/deep learning), region (China or other), and imaging modality (MRI, ultrasound, CT, PET–CT or MMG), was performed.

In addition, to assess the relative contribution of a single study to the overall estimate, sensitivity analyses were done by sequential exclusion of one study from the meta-analysis calculations. Publication bias was examined using Deek’s funnel plots, where slope coefficients with *P* < 0.10 indicated significant publication bias (20).

### Clinical utility

The clinical utility of radiomics in predicting LNM was evaluated with Fagan plot analysis by indicating the posttest probability (P post) of LNM when pretest probabilities (P pre, suspicion of LNM) were provided (21).

## Results

### Literature search

**Figure 1:**
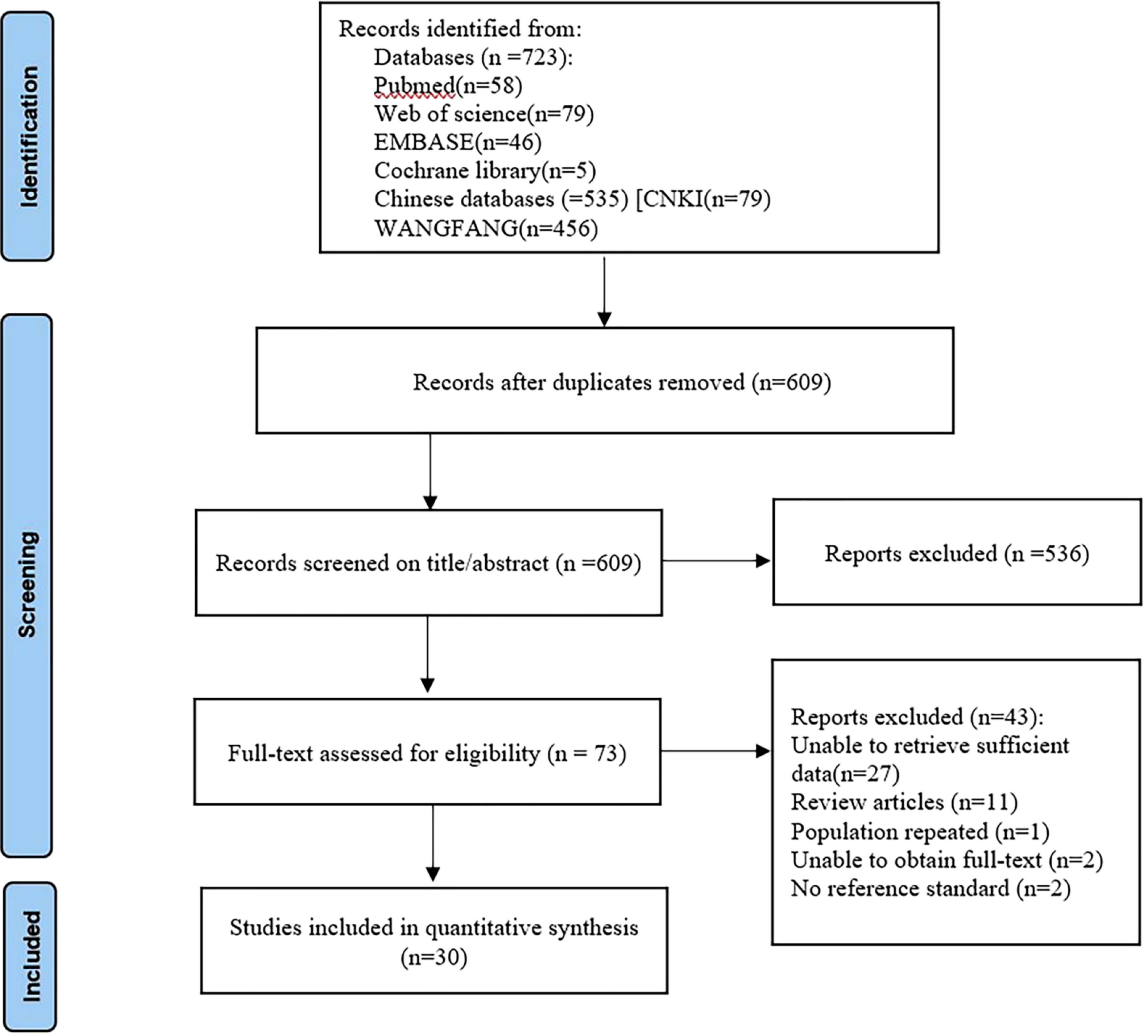
Flow diagram of study selection for meta-analysis according to PRISMA. PRISMA, Preferred Reported Items for Systematic Reviews and Meta analyses.

According to search strategies described in the methods section, 723 records were initially retrieved from the electronic databases. After removing 114 duplicates, 609 studies remained. They were screened by title and abstract for relevance, and 536 were excluded based on the inclusion and exclusion criteria. Finally, the 2 independent observers scrutinized 73 full-text articles and included 30 in the meta-analysis. **Figure 1** shows the flow chart that summarizes the study selection process.

### Data quality assessment

**Figure 2:**
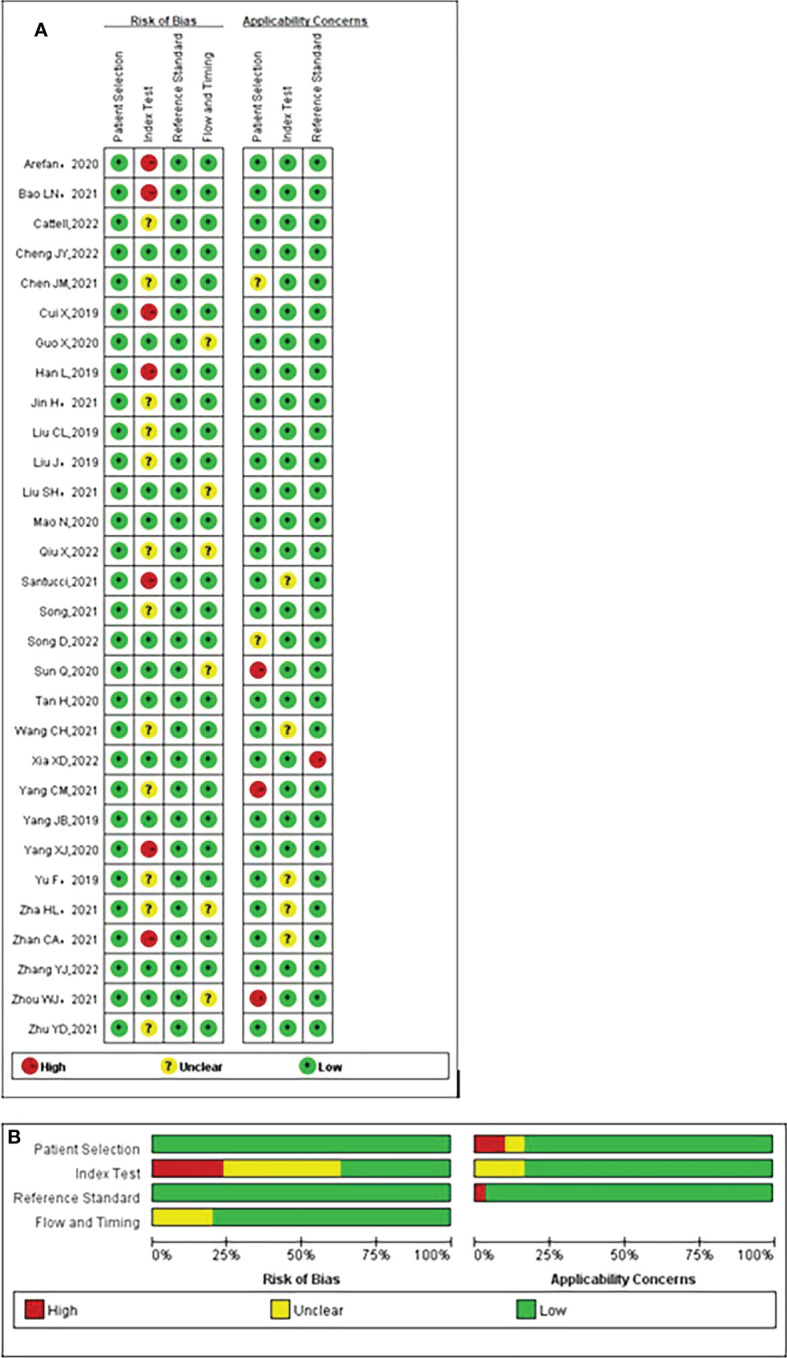
Quality assessment of included studies according to Quality Assessment of Diagnostic Accuracy Studies-2 (QUADAS-2) criteria. **(A)** Individual studies, **(B)** summary. Green, yellow, and red circles denote low, unclear, and high bias risks, respectively.

Overall, most studies exhibited very low risk of bias and few applicability problems, suggesting high data quality. The details of the risk of bias and applicability concerns of the included studies are presented in **Figure 2**.

### Characteristics of the included studies

**Table 1** shows relevant characteristics and details of 30 studies included in the meta-analysis. The studies had 5611 patients, 2337 with LNM and 3274 without, assessed using a radiomics method. Seven studies (3, 22–27) reported diagnostic performance based on radiological characteristics and clinical factors. Only one (10) used prospectively collected data. Furthermore, the studies applied radiomics based on different imaging modalities: MRI, 17 (3, 11, 22, 23, 28–39); ultrasound, 7 (14, 24, 25, 27, 40–42); CT, 2 (12, 43); PET–CT, 2 (10, 44); and MMG, 3 (13, 26, 45).

**Table 1:** The baseline characteristics of included studies.

study	study design	region	NO.patient	BC LNM	BC non-LNM	TP	FP	FN	TN	Reference standard	Imaging Modality	Radiomics algorithm	Combine Clinical Factors (Yes/No)
Arefan, 2020	retrospective	USA	154	80	74	58	16	22	58	pathology	MRI	machine learning	NO
Bao LN, 2021	retrospective	China	106	51	55	42	16	9	39	SLNB	US	Radiomic algorithm	NO
Cattell,2022	retrospective	USA	109	37	72	33	10	4	62	histopathology and SLNB	MRI	deep learning	YES
Chen JM,2021	retrospective	China	99	31	68	28	2	3	66	pathology	MRI	Radiomic algorithm	YES
Cheng JY,2022	prospective	China	203	109	94	87	29	22	65	histology	PET-CT	machine learning	NO
Cui X,2019	retrospective	China	115	52	63	49	13	3	50	SLNB or ALND	MRI	machine learning	NO
Guo X,2020	retrospective	China	542	180	362	175	152	5	210	SLNB or ALND	US	deep learning	NO
Han L,2019	retrospective	China	279	97	182	86	78	11	104	pathology	MRI	machine learning	NO
Jin H, 2021	retrospective	China	300	118	182	99	9	19	173	pathology	US	Radiomic algorithm	YES
Liu CL,2019	retrospective	China	109	37	72	28	13	9	59	histology	MRI	machine learning	NO
Liu J, 2019	retrospective	China	49	27	22	20	5	7	17	pathology	MRI	machine learning	NO
Liu SH, 2021	retrospective	China	100	55	45	49	5	6	40	pathology	MRI	Radiomic algorithm	NO
Mao N,2020	retrospective	China	270	143	127	136	95	7	32	histology	MMG	Radiomic algorithm	YES
Qiu X,2022	retrospective	China	71	39	32	38	10	1	22	pathology	MRI	Radiomic algorithm	NO
Santucci,2021	retrospective	Italy	99	72	27	62	7	10	20	histology	MRI	machine learning	NO
Song D,2022	retrospective	China	296	106	190	94	61	12	129	pathology	MRI	Radiomic algorithm	NO
Song,2021	retrospective	Korea	25	11	14	10	4	1	10	SLNB or ALND	PET-CT	machine learning	NO
Sun Q,2020	retrospective	China	359	101	258	94	19	7	239	histology	US	deep learning	NO
Tan H,2020	retrospective	China	269	96	173	81	48	15	125	SLNB or ALND	MRI	machine learning	NO
Wang CH,2021	retrospective	China	186	93	93	68	26	25	67	pathology	MRI	Radiomic algorithm	NO
Xia XD,2022	retrospective	China	168	89	79	80	5	9	74	pathology	MRI	Radiomic algorithm	YES
Yang CM,2021	retrospective	China	165	80	85	66	3	14	82	pathology	CT	machine learning	NO
Yang JB,2019	retrospective	China	110	61	49	51	8	10	41	pathology	MMG	machine learning	NO
Yang XJ,2020	retrospective	China	184	71	113	60	38	11	75	histology	CT	deep learning	NO
Yu F, 2019	retrospective	China	426	185	241	133	45	52	196	SLNB or ALND	US	Radiomic algorithm	YES
Zha HL, 2021	retrospective	China	318	92	226	78	57	14	169	pathology	US	Radiomic algorithm	YES
Zhan CA, 2021	retrospective	China	115	53	62	45	20	8	42	histology	MRI	machine learning	NO
Zhang YJ,2022	retrospective	China	130	58	72	42	21	16	51	pathology	MMG	Radiomic algorithm	NO
Zhou WJ, 2021	retrospective	China	132	57	75	45	17	12	58	pathology	US	Radiomic algorithm	NO
Zhu YD,2021	retrospective	China	123	56	67	40	4	16	63	histology	MRI	machine learning	NO

N, number of patients; BC, breast cancer; LNM, lymph node metastasis; TP, true positive; FP, false positive; TN, true negative; FN, false negative; US, ultrasound; CT, computed tomography; MRI, magnetic resonance imaging; MMG, mammography; PET-CT, positron emission tomography-computed tomography; SLNB, sentinel lymph node biopsy; ALND, axillary lymph node dissection.

### Data analysis

**Figure 3:**
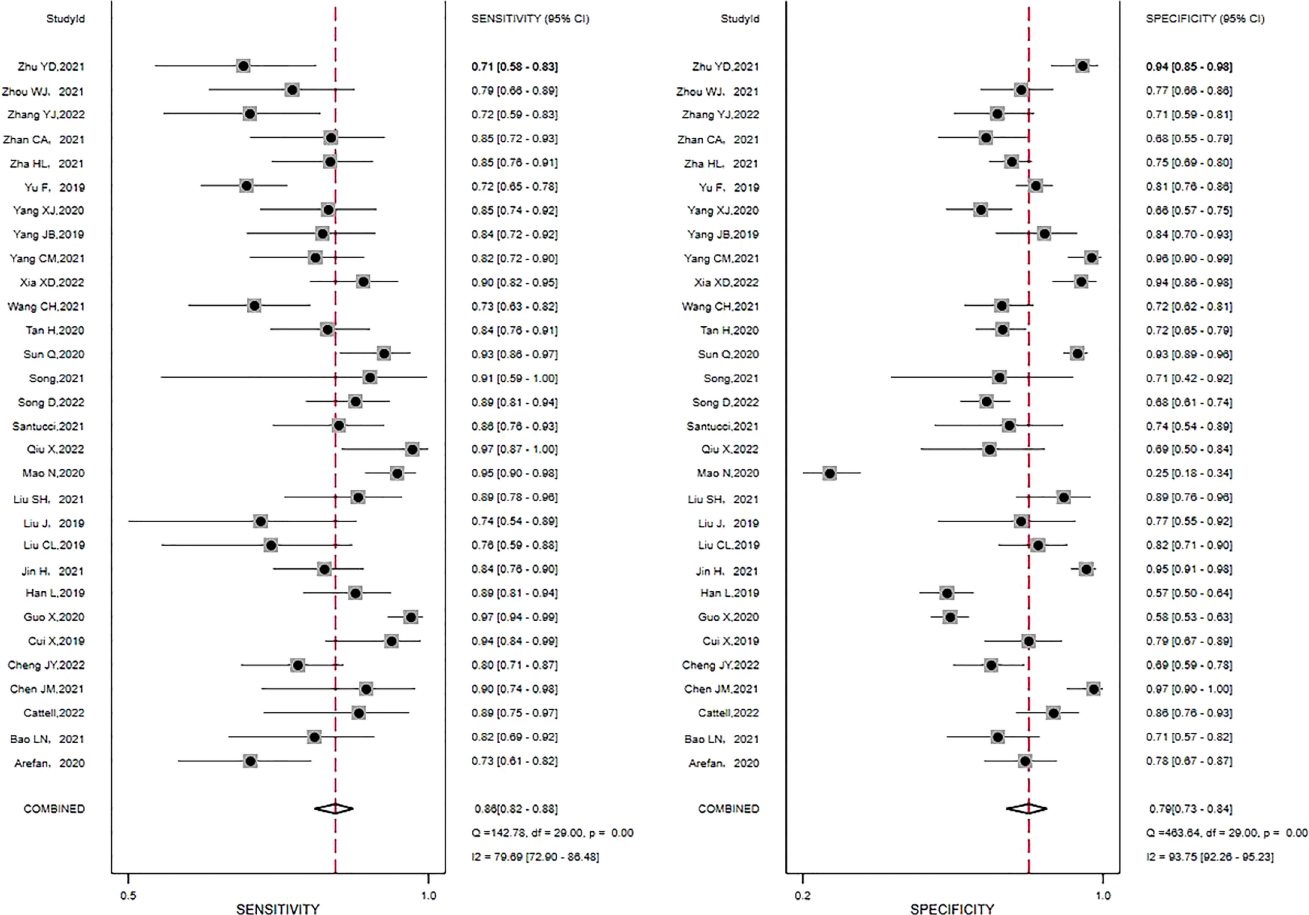
Forest plots of the sensitivity and specificity of radiomics in predicting ALNM in breast cancer. Numbers are pooled estimates, with 95% CIs depicted with horizontal lines. Heterogeneity statistics are shown at bottom right. *I^2^* >50% indicates substantial heterogeneity in the diagnostic parameters across studies.

**Figure 4:**
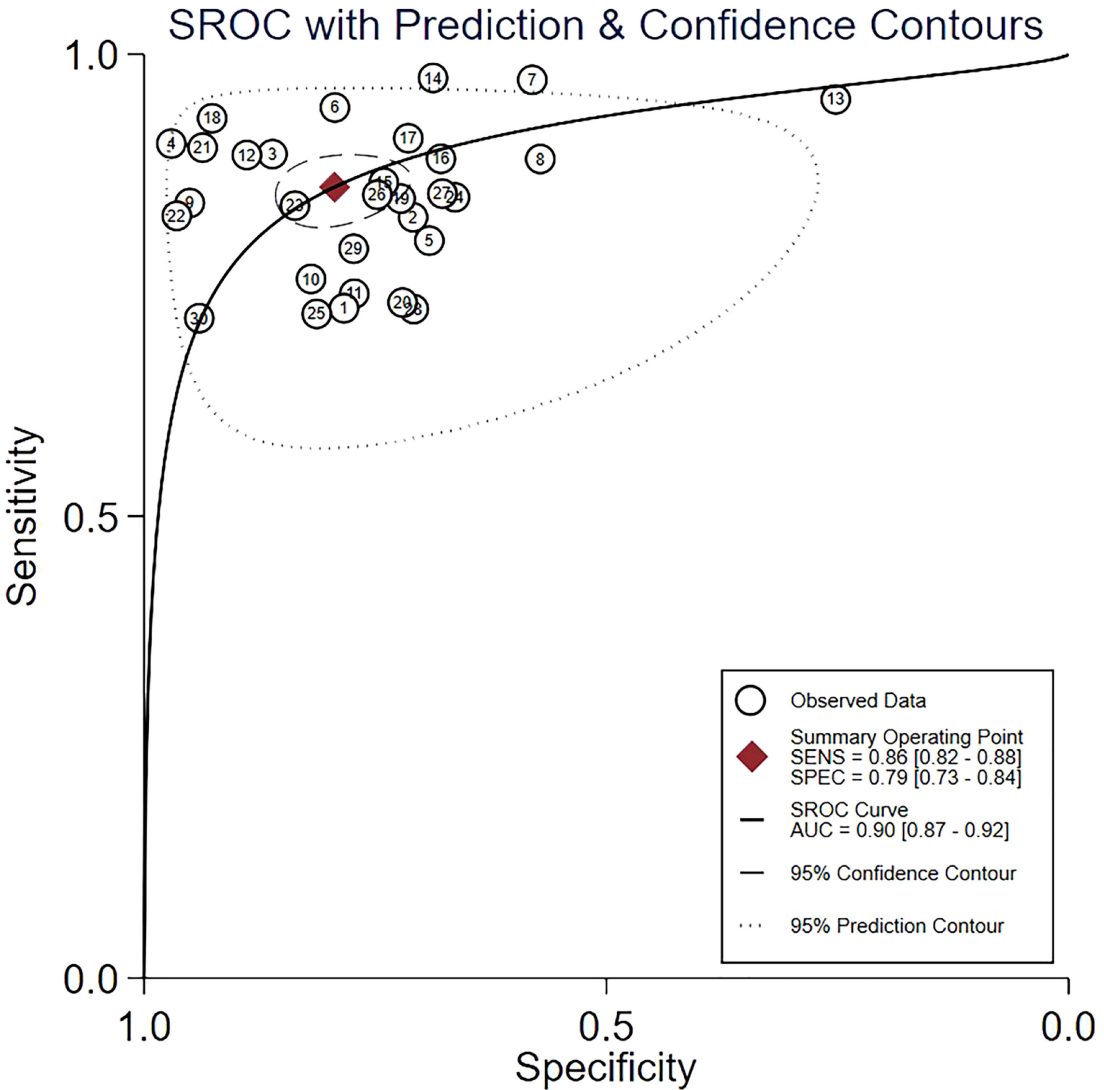
The summary receiver operating characteristic (SROC) curve of the diagnostic accuracy of radiomics in predicting ALNM in breast cancer. Each circle indicates one included study. Values in brackets are 95% CIs. AUC, area under the receiver operating characteristic curve.

Pooled estimates reflecting the overall predictive value of radiomic signatures for ALNM and SLNM in breast cancer were calculated across the studies: sensitivity, 0.86 (95% CI, 0.82-0.88); specificity, 0.79 (95% CI, 0.73-0.84); positive LR, 4.2 (95% CI, 3.2-5.4); negative LR, 0.18 (95% CI, 0.15-0.22); and DOR, 23 (95% CI, 16-33). In addition, an SROC curve was generated, and the AUC of the SROC curve was 0.90 (95% CI, 0.87-0.92), indicating a high overall diagnostic value. The forest plots for sensitivity and specificity are illustrated in **Figure 3**, and the SROC curve is presented in **Figure 4**.

### Heterogeneity estimation

The *I^2^* statistic revealed significant heterogeneity between sensitivity (*I^2^* = 79.69%) and specificity (*I^2^* = 93.75%) across the studies. Spearman’s correlation coefficient of 0.185 (*P* = 0.328) was calculated, implying the threshold effect does not exist.

Next, univariable meta-regression and subgroup analyses were performed to identify the source of heterogeneity. **Table 2** summarizes the univariable meta-regression and subgroup analyses results, suggesting the influence of study design, clinical factors, modeling methods, region, and specific imaging modality on the pooled sensitivity and specificity estimates.

**Table 2:** Univariable meta-regression and subgroup analyses.

Parameter	Category	No. of Studies	Sensitivity	P1	Specificity	P2
Study design	retrospective	29	0.86 (0.83,0.89)	0.65	0.80 (0.74,0.85)	0.63
	prospective	1	0.80 (0.61,0.99)		0.69 (0.31,1.00)	
Combine clinical factors	Yes	7	0.87 (0.82,0.93)	<0.01	0.84 (0.75,0.93)	0.15
	No	23	0.85 (0.82,0.89)		0.78 (0.71,0.84)	
Modeling methods	Radiomic algorithm	13	0.85 (0.81,0.90)	<0.01	0.79 (0.71,0.88)	0.01
	Machine/Deep learning	17	0.86 (0.82,0.90)		0.80 (0.72,0.87)	
Region	China	26	0.86 (0.83,0.89)	0.01	0.79 (0.74,0.85)	0.25
	Other countries	4	0.84 (0.75,0.94)		0.79 (0.63,0.95)	
MRI	Yes	16	0.85 (0.81,0.90)	<0.01	0.81 (0.74,0.88)	0.03
	No	14	0.86 (0.82,0.90)		0.78 (0.69,0.86)	
US	Yes	7	0.87 (0.81,0.92)	<0.01	0.82 (0.72,0.92)	0.08
	No	23	0.85 (0.82,0.89)		0.79 (0.72,0.85)	
CT	Yes	2	0.84 (0.72,0.96)	0.03	0.86 (0.71,1.00)	0.98
	No	28	0.86 (0.83,0.89)		0.79 (0.73,0.85)	
PET-CT	Yes	2	0.83 (0.69,0.98)	0.08	0.70 (0.42,0.99)	0.28
	No	28	0.86 (0.83,0.89)		0.80 (0.74,0.85)	
MMG	Yes	3	0.86 (0.78,0.95)	0.01	0.61 (0.37,0.84)	0.02
	No	27	0.86 (0.82,0.89)		0.81 (0.76,0.86)	

In terms of study design, retrospective studies (n = 29) had higher sensitivity 0.86 (95% CI, 0.83-0.89) and specificity 0.80 (95% CI, 0.74-0.85) than the prospective (n = 1; sensitivity, 0.80 [95% CI, 0.61-0.99]; specificity, 0.69 [95% CI, 0.31-1.00.]). The pooled sensitivity 0.87 (95% CI, 0.82-0.93) and specificity 0.84 (95% CI, 0.75-0.93) of radiomics combined with clinical factors (n = 7) were slightly higher than those using only radiomic features (n = 23; sensitivity, 0.85 [95% CI, 0.82-0.89]; specificity, 0.78 (95% CI, 0.71-0.84]). If a modeling method used radiomic algorithm or machine/deep learning, either showed similar sensitivity (0.85 [95% CI, 0.81-0.90] vs 0.86 [95% CI, 0.82-0.90]) and specificity (0.79 [95% CI, 0.71-0.88] vs 0.80 [95% CI, 0.72-0.87]). Similarly, region (China or other countries) yielded almost equivalent sensitivity (0.86 [95% CI, 0.83-0.89] vs 0.84 [95% CI, 0.75-0.94]) and specificity (0.79 [95% CI, 0.74-0.85] vs 0.79 [95% CI, 0.63-0.95]). In terms of different imaging modalities, 7 studies that used ultrasound had a higher sensitivity 0.87 (95% CI, 0.81-0.92) and specificity 0.82 (95% CI, 0.72-0.92) than MRI (n = 16; sensitivity, 0.85 [95% CI, 0.81-0.90]; specificity, 0.81 [95% CI, 0.74-0.88]), PET–CT (n = 2; sensitivity, 0.83 [95% CI; 0.69-0.98]; specificity, 0.70 [95% CI, 0.42-0.99]), and MMG (n = 3; sensitivity, 0.86 [95% CI, 0.78-0.95]; specificity, 0.61 [95% CI, 0.37-0.84]). Although the pooled sensitivity 0.84 (95% CI, 0.72-0.96) of the 2 CT studies was lower than that of ultrasound, the pooled specificity 0.86 (95% CI, 0.71-1.00) was higher. The source of heterogeneity between sensitivity and specificity across the studies varies depending on the tested covariate.

### Sensitivity analyses

No significant changes were observed in the overall heterogeneity among the included studies when one study was sequentially omitted from the analysis. Hence, these results agree with those of the main analyses and are shown in **Table 3**.

**Table 3:** Sensitivity analysis based on radiomics in predicting ALNM in breast cancer.

Eliminate study	Sensitivity	Specificity	PLR	NLR	DOR	AUC
Arefan, 2020	0.86 (0.83,0.89)	0.79 (0.73,0.85)	4.2 (3.2,5.5)	0.18 (0.14,0.22)	24 (16,35)	0.90 (0.87,0.92)
Bao LN, 2021	0.86 (0.82,0.89)	0.80 (0.73,0.85)	4.2 (3.2,5.5)	0.18 (0.15,0.22)	24 (16,34)	0.90 (0.87,0.92)
Cattell,2022	0.86 (0.82,0.88)	0.79 (0.73,0.84)	4.1 (3.1,5.4)	0.18 (0.15,0.23)	22 (15,33)	0.90 (0.87,0.92)
Chen JM,2021	0.86 (0.82,0.88)	0.78 (0.72,0.83)	4.0 (3.1,5.1)	0.18 (0.15,0.23)	21 (15,30)	0.89 (0.86,0.92)
Cheng JY,2022	0.86 (0.83,0.89)	0.80 (0.73,0.85)	4.2 (3.2,5.6)	0.18 (0.14,0.22)	24 (16,35)	0.90 (0.87,0.92)
Cui X,2019	0.85 (0.82,0.88)	0.79 (0.73,0.85)	4.1 (3.2,5.4)	0.19 (0.15,0.23)	22 (15,33)	0.90 (0.87,0.92)
Guo X,2020	0.85 (0.82,0.87)	0.80 (0.74,0.85)	4.2 (3.2,5.6)	0.19 (0.16,0.23)	22 (15,32)	0.89 (0.86,0.92)
Han L,2019	0.86 (0.82,0.88)	0.80 (0.74,0.85)	4.3 (3.3,5.6)	0.18 (0.15,0.22)	24 (16,35)	0.90 (0.87,0.92)
Jin H, 2021	0.86 (0.82,0.89)	0.78 (0.72,0.83)	4.0 (3.1,5.1)	0.18 (0.15,0.23)	22 (15,31)	0.90 (0.87,0.92)
Liu CL,2019	0.86 (0.83,0.89)	0.79 (0.73,0.85)	4.2 (3.2,5.5)	0.18 (0.14,0.22)	23 (16,34)	0.90 (0.87,0.92)
Liu J, 2019	0.86 (0.83,0.89)	0.79 (0.73,0.85)	4.2 (3.2,5.5)	0.18 (0.14,0.22)	23 (16,34)	0.90 (0.87,0.92)
Liu SH, 2021	0.85 (0.82,0.88)	0.79 (0.73,0.84)	4.1 (3.1,5.3)	0.18 (0.15,0.23)	22 (15,32)	0.90 (0.87,0.92)
Mao N,2020	0.85 (0.82,0.88)	0.81 (0.76,0.85)	4.4 (3.4,5.6)	0.19 (0.15,0.23)	24 (16,34)	0.90 (0.87,0.92)
Qiu X,2022	0.85 (0.82,0.88)	0.80 (0.73,0.85)	4.2 (3.2,5.5)	0.19 (0.15,0.23)	22 (15,33)	0.90 (0.87,0.92)
Santucci,2021	0.86 (0.82,0.88)	0.80 (0.73,0.85)	4.2 (3.2,5.5)	0.18 (0.15,0.22)	23 (16,34)	0.90 (0.87,0.92)
Song D,2022	0.86 (0.82,0.88)	0.80 (0.74,0.85)	4.2 (3.2,5.6)	0.18 (0.15,0.22)	23 (16,34)	0.90 (0.87,0.92)
Song,2021	0.86 (0.82,0.88)	0.80 (0.74,0.85)	4.2 (3.2,5.5)	0.18 (0.15,0.22)	23 (16,34)	0.90 (0.87,0.92)
Sun Q,2020	0.85 (0.82,0.88)	0.79 (0.72,0.84)	4.0 (3.1,5.1)	0.19 (0.15,0.23)	21 (15,30)	0.89 (0.86,0.92)
Tan H,2020	0.86 (0.82,0.89)	0.80 (0.73,0.85)	4.2 (3.2,5.5)	0.18 (0.15,0.22)	23 (16,34)	0.90 (0.87,0.92)
Wang CH,2021	0.86 (0.83,0.89)	0.80 (0.73,0.85)	4.2 (3.2,5.6)	0.18 (0.14,0.22)	24 (17,35)	0.90 (0.87,0.92)
Xia XD,2022	0.85 (0.82,0.88)	0.79 (0.72,0.84)	4.0 (3.1,5.2)	0.19 (0.15,0.23)	22 (15,31)	0.89 (0.86,0.92)
Yang CM,2021	0.86 (0.82,0.89)	0.78 (0.72,0.83)	4.0 (3.1,5.1)	0.18 (0.15,0.22)	22 (15,31)	0.90 (0.87,0.92)
Yang JB,2019	0.86 (0.82,0.89)	0.79 (0.73,0.84)	4.1 (3.1,5.4)	0.18 (0.15,0.22)	23 (16,34)	0.90 (0.87,0.92)
Yang XJ,2020	0.86 (0.82,0.88)	0.80 (0.74,0.85)	4.2 (3.2,5.6)	0.18 (0.15,0.22)	24 (16,35)	0.90 (0.87,0.92)
Yu F, 2019	0.86 (0.83,0.89)	0.79 (0.73,0.85)	4.2 (3.2,5.5)	0.18 (0.14,0.22)	24 (16,35)	0.90 (0.87,0.92)
Zha HL, 2021	0.86 (0.82,0.88)	0.80 (0.73,0.85)	4.2 (3.2,5.5)	0.18 (0.15,0.22)	23 (16,34)	0.90 (0.87,0.92)
Zhan CA, 2021	0.86 (0.82,0.88)	0.80 (0.74,0.85)	4.2 (3.2,5.6)	0.18 (0.15,0.22)	24 (16,34)	0.90 (0.87,0.92)
Zhang YJ,2022	0.86 (0.83,0.89)	0.80 (0.73,0.85)	4.2 (3.2,5.6)	0.18 (0.14,0.22)	24 (17,35)	0.90 (0.87,0.92)
Zhou WJ, 2021	0.86 (0.83,0.89)	0.79 (0.73,0.85)	4.2 (3.2,5.5)	0.18 (0.14,0.22)	23 (16,34)	0.90 (0.87,0.92)
Zhu YD,2021	0.86 (0.83,0.89)	0.79 (0.72,0.84)	4.0 (3.1,5.2)	0.18 (0.14,0.22)	23 (15,33)	0.90 (0.87,0.92)
overall	0.86 (0.82,0.88)	0.79 (0.73,0.84)	4.2 (3.2,5.4)	0.18 (0.15,0.22)	23 (16,33)	0.90 (0.87,0.92)

SEN, sensitivity; SPE, specificity; PLR, positive likelihood ratio; NLR, negative likelihood ratio; DOR, diagnostic odds ratio; AUC, area under the curve.

### Publication bias

**Figure 5:**
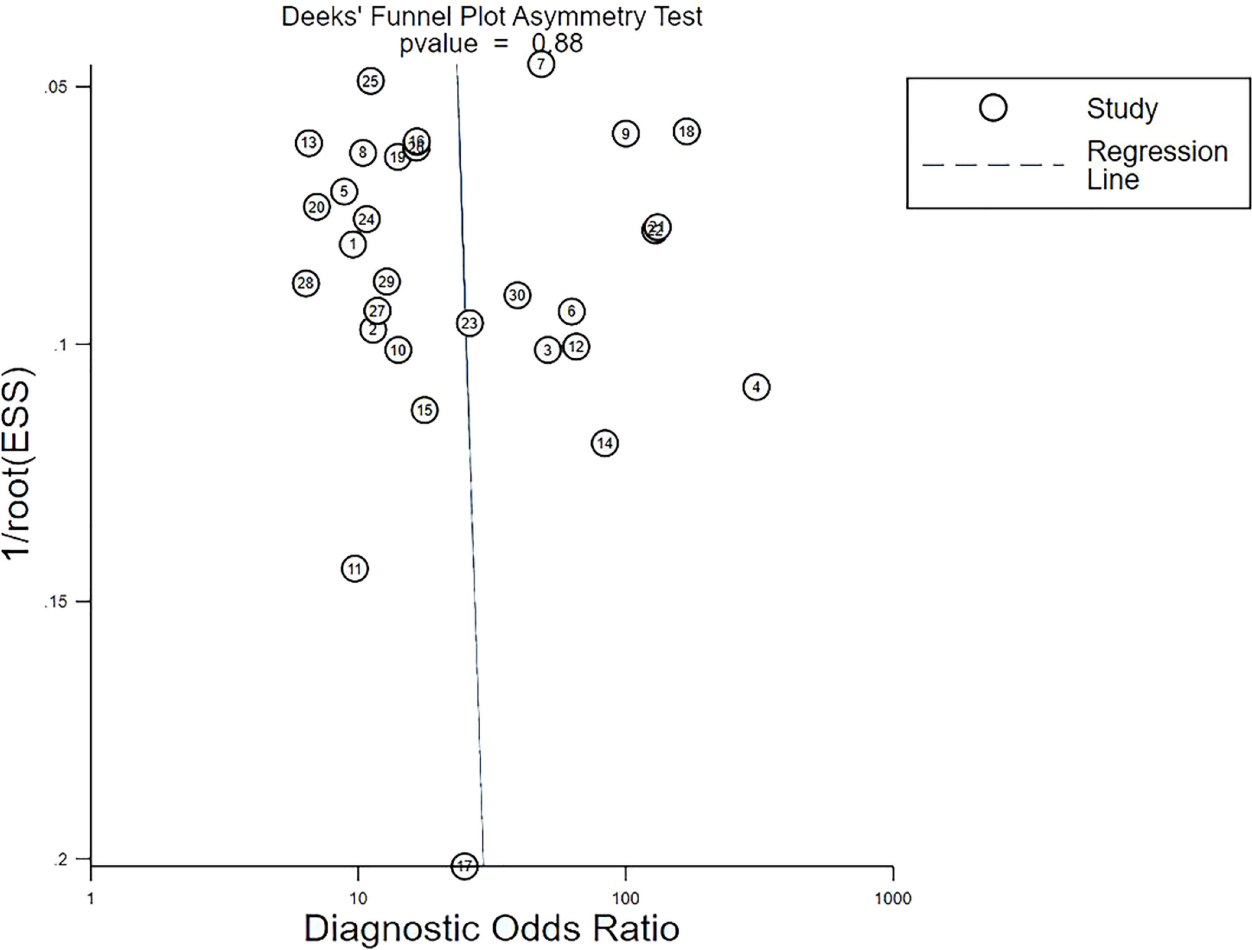
Deeks’ funnel plot asymmetry test for publication bias. Deeks’ funnel plot shows no asymmetry and the presence of publication bias. Each circle indicates one included study. ESS, effective sample size.

Deek’s funnel plot revealed a high slope coefficient (*P* = 0.88) (**Figure 5**), suggesting the absence of publication bias among the studies.

### Clinical utility

**Figure 6:**
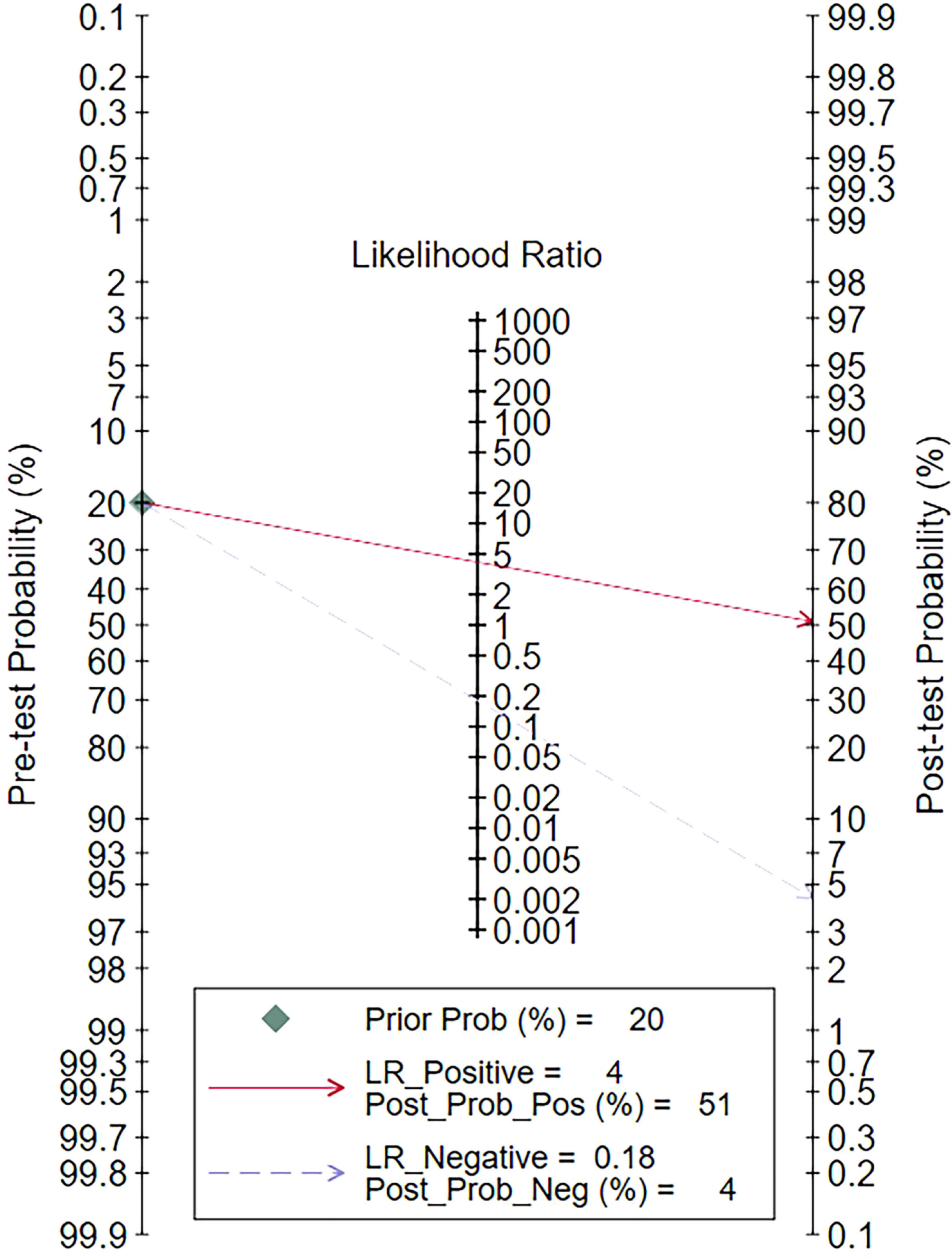
Fagan plots for assessing the clinical utility. LR, likelihood ratio; Prob, probability; Pos, positive; Neg, negative.

Using radiomic features based on various imaging modalities would increase the posttest probability to 51% from 20% with a PLR of 4 when the pretest was positive and would reduce the posttest probability to 4% with an NLR of 0.18 when the pretest was negative (**Figure 6**).

Radiomic features based on various imaging modalities increased the posttest probability to 51% from 20%, with a positive LR of 4 when the pretest was positive. Conversely, they reduced the posttest probability to 4%, with a negative LR of 0.18 when the pretest was negative (**Figure 6**).

## Discussion

In our systematic review with meta-analysis, we determined pooled estimates of sensitivity 0.86 (95% CI, 0.82-0.88), specificity 0.79 (95% CI, 0.73-0.84), and AUC 0.90 (95% CI, 0.87-0.92). These values indicate that radiomics is an effective and accurate tool for predicting ALNM and SLNM in breast cancer, which will help clinicians select safe and effective treatments for patients and reduce postoperative complications.

Sensitivity analyses were performed on the 30 included studies with a sequential exclusion approach. Despite omitting a study with each step, pooled sensitivity and specificity were unaffected, and radiomics continued to show good predictive ability for ALNM and SLNM. Furthermore, Deek’s funnel plot pointed to the absence of published bias, suggesting our results are reliable. Likelihood ratios and posttest probabilities are indexes of the diagnostic tests and provide crucial information about the likelihood that a patient with a positive or negative test has LNM. Radiomic features increased the posttest probability to 51% from 20%, with a positive LR of 4 when the pretest was positive. By contrast, the signatures reduced the posttest probability to 4%, with a negative LR of 0.18 when the pretest was negative. These data demonstrate that radiomics has important clinical value in improving the accuracy of predicting LNM in patients with breast cancer.

Heterogeneity is common in meta-analysis. In our study, we observed significant heterogeneity between the studies, which could have been caused by threshold effect, study design, or even imaging modality. Spearman’s correlation coefficient value was insignificant, eliminating the threshold effect as the potential source of heterogeneity in our meta-analysis. Therefore, meta-regression and subgroup analyses were performed to identify the source of heterogeneity, but due to the limited number of included studies, univariate meta-regression analyses were performed instead of multivariate meta-regression analyses. Differences in study design and region can provide different information and research quality and cause heterogeneity. Various clinical factors combined with radiomics confer high diagnostic efficiency. However, they can also be a source of heterogeneity. Furthermore, different modeling methods and imaging modalities also create heterogeneity (10–14). Although previous studies considered different sources of heterogeneity, a systematic study of the radiomics performance in predicting ALNM and SLNM in breast cancer was lacking. Therefore, we used 5 parameters for univariate meta-regression and subgroup analysis to identify the probable sources of heterogeneity to help address the gap.

Subgroup analysis revealed that although retrospective studies were superior to the prospective, their results were insignificant. Because most of the studies in this meta-analysis were retrospective (29 out of 30), they are prone to selection bias. Prospective studies result in better research quality due to their completeness. In addition, since exposure is evaluated before the outcome, they are less prone to bias. Therefore, future prospective studies should improve the predictive performance of radiomics for the evaluation of LNM in patients with breast cancer and its clinical efficiency. Our meta-analysis also showed that studies using radiomics combined with clinical factors have higher diagnostic performance than those relying only on radiomics, which is consistent with the previous studies (3, 25, 26). Thus, adding clinical features to radiomic imaging improves the accuracy of diagnosing LNM in breast cancer.

Subgroup analysis also uncovered that studies conducted in China had better diagnostic performance than those done abroad. However, this meta-analysis included only 4 studies from outside China, underscoring the need for more overseas studies to confirm the above conclusion. Modeling methods using radiomics algorithm and machine/deep learning had a similar diagnostic performance. This result opposes previous evidence that suggests that machine/deep learning is superior to the traditional radiomics algorithm in predicting LNM in breast cancer (3, 39, 42) and requires further verification.

Finally, we performed a subgroup analysis of the diagnostic performance of radiomics with different imaging modalities. Although ultrasound had the highest diagnostic performance, only 7 ultrasound studies were included, of which 5 combined with clinical factors or deep learning algorithms. Therefore, the pooled results do not entirely prove that ultrasound has the highest diagnostic performance. Moreover, this modality is susceptible to operator subjectivity (46). The radiomics model based on feature extraction of preoperative MRI images also had a high predictive performance. This result is similar to a previous study that used dynamic contrast-enhanced–MRI (DCE–MRI) radiomic signatures of patients with breast cancer to predict LNM with satisfactory results (47). The pooled sensitivity of MMG was similar to that of ultrasound and MRI, but pooled specificity was lower (only 0.61). Only 4 included studies combined radiomics with CT or PET–CT. Hence, the statistical differences calculated with the subgroup analysis were insignificant, and more studies with these modalities should verify their diagnostic performance. Although this meta-analysis addresses various sources of heterogeneity, other unmentioned differences between the studies may have also contributed to heterogeneity.

This study has several advantages. First, to our knowledge, it is the first meta-analysis that comprehensively evaluates the diagnostic test accuracy of radiomics models in predicting ALNM and SLNM in breast cancer. Before it, a meta-analysis (48) of DCE –MRI based on machine learning also had a good diagnostic performance, with an AUC of 0.89, but it was only a part of radiomics. Second, this study considers different imaging modalities and modeling methods, providing novel ideas for subsequent radiomics research.

We also acknowledge that our meta-analysis has certain limitations. We mainly included retrospective studies and only 1 prospective, which makes our study prone to patient selection bias and data loss. Therefore, more prospective trials are necessary to validate our findings. In addition, we found significant heterogeneity in the pooled sensitivity and specificity. This observation is similar to that in published meta-analyses of diagnostic accuracy using radiomics (49–52). Although radiomic models help predict the diagnosis of LNM in breast cancer, they involve numerous analysis methods. Thus, the choice of imaging modality and modeling method may affect the predictive results of radiomics analysis, causing heterogeneity. Furthermore, during data extraction, we selected the model with the highest diagnostic performance among multiple, which might have overestimated the pooled sensitivity and specificity of radiomics for LNM in breast cancer. Finally, most of the included studies were performed in China, which might have affected the general applicability of the results in clinical practice.

## Conclusion

In conclusion, our meta-analysis demonstrates that radiomic models based on preoperative imaging features have good diagnostic performance in predicting ALNM and SLNM in patients with breast cancer. Radiomics is a promising noninvasive method and is expected to provide new quantitative diagnostic techniques for clinical practice. Future well-designed radiomics experiments are needed to verify its effectiveness and diagnostic performance, reduce its heterogeneity, and enable its wide clinical application.

## Data availability statement

The original contributions presented in the study are included in the article/supplementary material. Further inquiries can be directed to the corresponding author.

## Author contributions

Conception and study design: XG, YG, and MZ. Literature search and study selection: XG, YG, and TZ. Data acquisition and quality assessment: TZ and XP. Data analysis: XG, YG, TZ, and XP. Study supervision: DX and MZ. Manuscript writing: XG, YG, TZ, XP, DX, and MZ. Administrative support: DX and MZ. All authors contributed to the article and approved the submitted version.

## Funding

This work was supported by Natural Science Foundation of Shanghai (Research Grant No.19ZR1452400) and National Natural Science Foundation of China (Research Grant No. 81673743).

## Conflict of interest

The authors declare that the research was conducted in the absence of any commercial or financial relationships that could be construed as a potential conflict of interest.

## Publisher’s note

All claims expressed in this article are solely those of the authors and do not necessarily represent those of their affiliated organizations, or those of the publisher, the editors and the reviewers. Any product that may be evaluated in this article, or claim that may be made by its manufacturer, is not guaranteed or endorsed by the publisher.
